# Botulinum Toxin in the Therapy of Chronic or Relapsing Plantar Fasciitis—A Descriptive Review

**DOI:** 10.3390/biomedicines13112647

**Published:** 2025-10-28

**Authors:** Daniela Poenaru, Claudia-Gabriela Potcovaru, Andreea Dumitrescu, Livia Alexandra Ion, Roxana Nartea, Delia Cinteza

**Affiliations:** 1 Rehabilitation Department, Carol Davila University of Medicine, 050474 Bucharest, Romania; daniela.poenaru@umfcd.ro (D.P.); delia.cinteza@umfcd.ro (D.C.); 2 Rehabilitation Department, National Institute of Rehabilitation, 030167 Bucharest, Romania; andreea.dumitescu@inrmfb.ro (A.D.); livia-alexandra.ion@rez.umfcd.ro (L.A.I.); roxana.nartea@umfcd.ro (R.N.)

**Keywords:** botulinum toxin, plantar fasciitis, intrafascial, intramuscular

## Abstract

**Background.** Plantar fasciitis is a degenerative condition that may follow a chronic or recurrent course. Injectable agents are frequently employed to alleviate pain and restore function. Recently, botulinum toxin (BoNT) has gained particular attention for its analgesic properties, positioning it as a promising therapeutic option for the management of plantar fasciitis. **Method**. We conducted a literature review to evaluate studies on BoNT administration in plantar fasciitis, aiming to define the target tissues, optimal doses, methods of administration, and associated adverse effects. **Results**. The evidence suggests that BoNT provides significant short- and long-term analgesic benefits when administered either intrafascially or intramuscularly, with outcomes comparable to or exceeding those of corticosteroids. **Conclusion**. Further high-quality studies are warranted to compare BoNT with other therapeutic modalities, such as PRP and extracorporeal shockwave therapy (ESWT).

## 1. Introduction

Plantar fasciitis is a degenerative condition affecting the plantar fascia at its insertion on the medial calcaneal tuberosity. Despite the terminology suggesting an inflammatory process, histopathological studies have not demonstrated the presence of inflammatory cells. The exact pathophysiology remains incompletely understood; however, several factors have been associated with its development, including repetitive microtrauma, elevated body mass index, prolonged weight-bearing activities related to occupation or lifestyle, and certain congenital anatomical variations. These factors contribute to repetitive mechanical stress, ultimately leading to micro-tears within the plantar fascia [1]. The condition may be present in young and active individuals as well as in old and sedentary people. Diabetic patients are at risk to develop this condition [2].

Clinically, plantar fasciitis is the most common cause of plantar heel pain syndrome. The diagnosis is based on patient description and clinical examination and various imagistic modalities sustain the alteration of the fascia [3]. In this context, adjacent neural structures may also be implicated. Specifically, compression of the medial and plantar nerve complex of the heel can occur between the quadratus plantae and/or abductor hallucis muscles. This may involve one or a combination of the medial plantar nerve, lateral plantar nerve, first branch of the lateral plantar nerve (or its variants), as well as the dense neuro-ganglia located within the quadratus plantae near its calcaneal origin. There is a consistent anatomical proximity between these medial heel nerves and the abductor hallucis and/or quadratus plantae muscles [4].

Plantar fascia is part of a functional and anatomical continuum encompassing the gastrocnemius–soleus complex, Achilles tendon, and calcaneus. Singh et al. (2021) [5] demonstrated strong anatomical correlations between Achilles tendon and plantar fascia at the calcaneus. Huerta et al. (2014) [6] described the mechanical influence of calf muscle tightness on plantar fascia strain. Clinical guidance and surgical strategies (e.g., gastrocnemius recession) prove this interconnected system aimed at reducing biomechanical overload and heel pain. Shortening or increased tension in any part of this continuum can increase mechanical load and pain across the system. In the same way, stretching of plantar fascia as a therapeutic approach is incorporated into a more extended stretching program including triceps surae and Achilles tendon.

The therapeutic approach to plantar fasciitis typically begins with conservative management, including relative rest, use of orthotic shoe inserts, night splints, physical modalities such as ultrasound and laser therapy, stretching exercises, and extracorporeal shockwave therapy (ESWT). Local injections may involve corticosteroids, platelet-rich plasma (PRP), or botulinum toxin (BoNT). Refractory or recurrent cases may warrant surgical intervention. Overall, conservative treatments are successful in approximately 80–90% of cases [7].

Botulinum neurotoxin (BoNT) is a protein produced by *Clostridium botulinum*. It targets specific proteins known as SNAREs (soluble N-ethylmaleimide-sensitive factor attachment protein receptors), which mediate neuronal exocytosis, thereby reversibly inhibiting the presynaptic release of neurotransmitters at the neuromuscular junction. The primary neurotransmitters affected are acetylcholine and substance P—a neuropeptide involved in neurotransmission and modulation of pain perception [8]. The reduction in acetylcholine results in temporary muscle weakness, while decreased substance P contributes to analgesic effects. Beyond its well-established paralytic action, the analgesic properties of BoNT are currently being investigated to potentially broaden its therapeutic indications [9]. The consequences of substance P release inhibition are the increase in peripheral threshold for the action potential, responsible for peripheral sensitization and the modulation of spinal transmission of the impulses, responsible for central sensitization.

**Figure 1:**
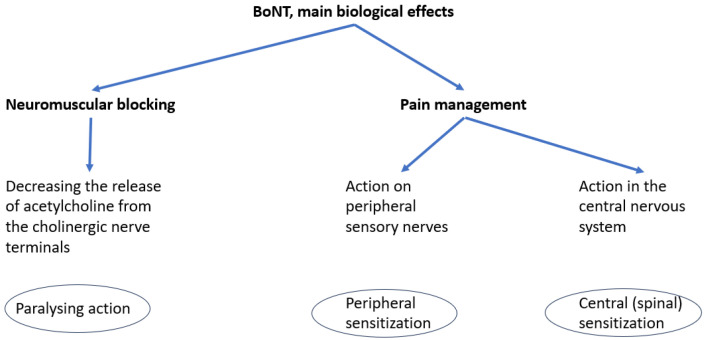
Main biological effects of BoNT.

In a previous paper, we figured a scheme of BoNT biological effects, see Figure 1 [10].

The research included studies involving patients with a diagnosis of plantar fasciitis, either in chronic or recurrent forms, who received botulinum toxin injections. Study selection was based on the route of administration, dosage, target tissues, and reported adverse effects. The role of botulinum toxin in the management of plantar fasciitis, as well as the optimal methodology of its administration, remains to be determined.

A literature search was conducted in PubMed/MEDLINE, Web of Science, Embase, and the Cochrane Library using the following MeSH terms and keywords: “plantar fasciitis” OR “plantar fasciopathy” AND “botulinum toxin.” The search was conducted from inception to March 2025. Inclusion criteria encompass all types of clinical trials, pilot studies, and case reports involving adult patients with clinically diagnosed chronic or recurrent plantar fasciitis with failure of previous therapies, treated with any type of BoNT. Exclusion criteria comprised animal studies, in vitro studies, systematic reviews, and meta-analyses.

Study selection was conducted by the team as follows: initially, two authors screened 2014 titles; following the removal of duplicates, 1872 titles were manually assessed against exclusion criteria. Eighteen full-text articles were subsequently reviewed. Among these, two articles reported on the same trial, and another two articles presented different phases of a single trial. After accounting for these overlaps, 16 unique studies were included in the final analysis.

**Figure 2:**
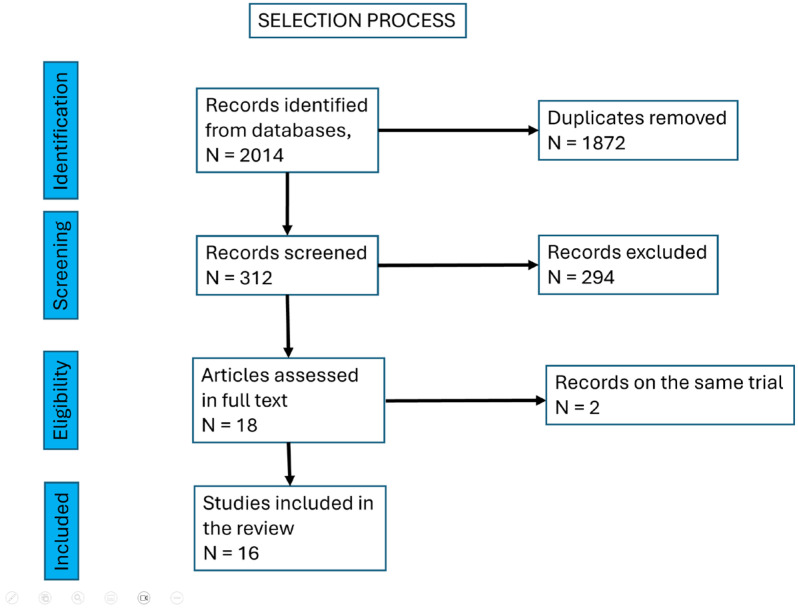
The selection process of the articles included.

The selection process is depicted in Figure 2.

## 2. Relevant Sections

A total of 13 randomized controlled trials [11,12,13,14,15,16,17,18,19,20,21,22,23], 2 pilot studies [24,25], and 1 case report [26] were identified. It is important to note that all included studies incorporated an exercise program, predominantly consisting of stretching exercises, as part of the intervention. Across these studies, a cumulative total of 725 patients with plantar fasciitis were enrolled as we see in Table 1.

The studies involved patients with chronic plantar fasciitis, with an evolution of pain and dysfunction for over 3 months. Two studies included patients with lower duration of symptoms (6 weeks and 10 weeks) [13,14]. Researchers used type A BoNT, with three formulations: onabotulinumtoxin A (Botox, Allergan), abobotulinumtoxinA (Dysport, Medicis), and incobotulinumtoxin A (Xeomin, Merz). The conversion ratios of these forms are mainly: onabotulinum:incobotulinum:abobotulinum = 1:1:2.5(3) [28].

In 2005 were reported the first attempts to treat refractory plantar fasciitis with BoNT, in patients with chronic evolution of 6 months. Babcock ret al. [11] administered a total of 70 units of onabotulinumtoxin A, divided into two injections: 40 units were delivered to the most tender point on the medial heel, targeting the plantar fascia origin, and 30 units were injected into the most tender area of the foot arch, aiming at the adductor hallucis and flexor digitorum brevis muscles. Injections were performed using palpation-based anatomical guidance. Patients in the BoNT group demonstrated statistically significant improvements in both pain and functional outcomes at 3 and 8 weeks compared to the placebo group (saline injection). Since this initial study, the injection technique has become associated with the name of the lead author, and a series of subsequent trials have adopted the same method. One case report monitored changes in pain intensity, pressure pain threshold (PPT), and plantar fascia thickness via ultrasound over a 7-week period. The authors observed improvement in pain and PPT during the first 3 to 5 weeks, followed by a gradual decline up to week 7, though values remained improved relative to baseline [26].

In a pilot study (9 patients, 4 months of chronic evolution), Placzek et al. administered 200 units of abobotulinumtoxinA via subfascial injection at the site of maximal pain near the origin of the plantar fascia. The procedure was performed under palpatory guidance, with the needle redirected in four different orientations to enhance distribution. Pain intensity was assessed in various contexts (e.g., within the past 48 h, at rest) over a 52-week follow-up. A reduction in pain was observed in the first 2 weeks, with sustained improvement noted at 14 weeks and still detectable at 52 weeks [24]. Subsequently, the authors conducted a randomized, placebo-controlled trial involving 33 patients using the same injection technique. Pain scores and pressure pain threshold (PPT) were evaluated over an 18-week period. Although patients in the BoNT group exhibited greater improvement in pain by week 6, the difference did not reach statistical significance. By week 18, no significant differences were observed between the treatment and placebo groups [12].

Ultrasound guidance, as a precise technique of assessing the plantar fascia, became widely used in the manipulation of botulinum toxin into the plantar fascia. In a randomized, placebo-controlled trial involving 50 patients, a single dose of 50 units of onabotulinumtoxin A was injected under ultrasound guidance. Outcomes assessed included pain intensity, plantar fascia thickness, and center of pressure (COP) velocity during the loading response phase, with follow-up extending to 3 months. The study demonstrated significant reductions in both pain and plantar fascia thickness at 3 weeks and at 3 months post-injection. Additionally, COP velocity in the affected foot increased, reaching values comparable to those of the asymptomatic contralateral foot [13].

### 2.1. Short Plantar Muscles

The target short plantar muscles included the abductor hallucis, flexor digitorum brevis and quadratus plantae. The rationale for targeting these muscles was to decompress the plantar and calcaneal nerves, thereby addressing the neuropathic component of plantar heel pain. Accurate localization and injection of these muscles can be achieved using ultrasound guidance or electrical stimulation techniques.

In a pilot study involving four patients (with symptoms duration from 3 months to 3 years), 50 units of abobotulinumtoxin A were injected into both the abductor hallucis and quadratus plantae muscles under electrical stimulation guidance. Pain, functional status, and disability were assessed over a 26-week follow-up period. Significant improvements were seen at 6 weeks and maintained through 24 weeks [24].

In a randomized, placebo-controlled trial involving 50 patients (with an average duration of symptoms of 18,8 weeks), 100 units of incobotulinumtoxin A were injected into the flexor digitorum brevis muscle under electrical stimulation guidance. The study reported significant improvements in both pain and functional outcomes at 6- and 12-month post-injection [14].

### 2.2. Triceps Injection

The rationale for injecting the triceps surae or its individual components is based on the observation that both acute and chronic plantar fasciitis are frequently associated with limited ankle dorsiflexion. In one study, 83% of patients with plantar fasciitis demonstrated restricted dorsiflexion, of whom 57% had an isolated gastrocnemius contracture, while 26% had a combined gastrocnemius–soleus complex contracture [29]. Anatomically, there is a continuity between the gastrocnemius–soleus complex, the Achilles tendon paratenon, and the plantar fascia. Consequently, increased tension in the Achilles tendon secondary to calf muscle tightness leads to elevated tension within the plantar fascia [4].

In a randomized, placebo-controlled trial involving 32 patients, 70 units of BoNT or saline were injected into the medial head of the gastrocnemius under ultrasound guidance. Pain, functional outcomes, and patient satisfaction were assessed over a 12-month follow-up period. The BoNT group demonstrated significant intragroup improvements in pain and function, with intergroup analysis showing superior results compared to placebo. Patients treated with BoNT experienced improved ankle dorsiflexion range of motion and were able to fully weight-bear and wear regular footwear immediately after treatment, whereas the placebo group achieved this at a mean of 12.6 weeks. Patient satisfaction was significantly higher in the BoNT group. No adverse effects or reduction in muscle strength were observed [15].

The summary of the doses and the routes of administration in the selected papers are presented in Table 2.

### 2.3. Comparative Studies

Three studies compared botulinum toxin (BoNT) with corticosteroid injections for the treatment of chronic plantar fasciitis (over 3 months symptoms). The injection techniques varied across the studies: the Babcock technique (70 units of onabotulinumtoxin A, 56 patients) [4,16], intrafascial ultrasound-guided injection (100 units of abobotulinumtoxinA, 50 patients) [17], and palpation-guided injection (50 units of onabotulinumtoxin A at the most tender point on the medial heel, 100 patients) [18]. In the short term (1 month), all trials demonstrated significant intragroup improvements in pain, function, and fascia thickness for both BoNT and corticosteroid groups, with no significant differences between the two. Starting at 4 months, clinical outcomes in the corticosteroid group decreased, while the BoNT group showed sustained levels of pain relief, function, and fascia thickness for up to 12 months.

Additional insight is provided by a study involving 71 patients with chronic fasciitis (over 2 months) randomized into three groups: local anesthetic, corticosteroid, and BoNT. The BoNT group received 200 units of abobotulinumtoxinA injected into the plantar fascia under ultrasound guidance, with the needle positioned subfascially near the fascia insertion and advanced in a fan-shaped pattern. Pain and functional outcomes were assessed over 6 months. All groups demonstrated significant improvement, with no statistically significant differences observed between them [20].

In a randomized controlled trial involving 53 patients, botulinum toxin (BoNT) was compared with platelet-rich plasma (PRP) injections. BoNT demonstrated significantly greater improvements in pain, function, and plantar fascia thickness at 1 month, whereas PRP showed superior outcomes at 12 months [19].

Administration of BoNT into the short plantar muscles or the calf muscles has been compared to traditional corticosteroid injections into the plantar fascia, yielding encouraging clinical outcomes.

A randomized, single-blind comparative trial involving 35 patients evaluated corticosteroid injection into the plantar fascia versus BoNT injection into the flexor digitorum brevis and quadratus plantae muscles under ultrasound guidance. Pain, functional outcomes, and plantar fascia thickness were assessed at 3 weeks, 12 weeks, and 6 months. At 3 weeks, both groups demonstrated significant improvement. However, the effects of corticosteroid injection diminished over time, whereas the BoNT group continued to improve through 12 weeks and maintained these benefits at 6 months [21].

A randomized controlled trial involving 36 patients (chronic fasciitis for 14 months) compared BoNT injections into the triceps surae muscle group (100 units in each gastrocnemius and 50 units in soleus muscle) with corticosteroid injections into the plantar fascia administered by palpation-guided technique. Both groups participated in a stretching program. Outcomes were assessed using pain scores and functional measures. In the short term (15 days), both groups showed improvement in pain and function. However, at medium- and long-term follow-up (up to 6 months), the BoNT plus stretching group demonstrated significantly greater improvements in both pain and function compared to the corticosteroid group [22].

Extracorporeal shockwave therapy (ESWT) is an established effective treatment for tendinopathies. A randomized controlled trial involving 72 patients (chronic plantar fasciitis for at least 6 months) compared a single session of focused ESWT to a single injection of 70 units of onabotulinumtoxin A administered using the Babcock technique. Both groups demonstrated significant intragroup improvements; however, the ESWT group showed superior outcomes during the follow-up visit between 1 and 2 months, suggesting that ESWT may be a more appropriate treatment modality for chronic plantar fasciitis [23].

## 3. Discussion

Plantar fasciitis is a pathological condition that resolves in a significant proportion of patients with conservative management. However, in chronic or recurrent cases, additional interventions may be required, including corticosteroid, BoNT, or PRP injections [4,30]. This paper focuses on the use of BoNT as an analgesic therapy, doses, modalities of injections and potential adverse effects.

It is important to note that injectable therapies were administered as part of a comprehensive rehabilitation program, which also included physical exercise and stretching as key components.

The techniques of botulinum toxin administration varied across the selected studies and included intrafascial injection, intramuscular injection either into the short plantar muscles or into the gastrocnemius–soleus complex, as well as combined injections into the fascia and adjacent short plantar muscles. Doses of 70 units of onabotulinumtoxin A or 200 units of abobotulinumtoxinA were reported. For onabotulinumtoxin A, the combined approach—40 units injected into the fascia and the remainder into the short plantar muscles under palpatory guidance—was initially validated by Babcock and has subsequently been widely adopted in studies attributed to his group.

Comparative analyses with corticosteroid injections demonstrated similarly favorable short-term outcomes (up to one month) but significantly superior long-term results (up to one year) for botulinum toxin. An additional advantage of botulinum toxin is its favorable safety profile, whereas corticosteroid injections are associated with a higher risk of adverse effects, including fascial rupture, fat pad atrophy, and cutaneous atrophy.

Intramuscular injection of botulinum toxin (BoNT) was performed either into the short plantar muscles or into the calf muscles and was associated with improvements in pain, function, and plantar fascia thickness when compared with placebo or corticosteroid injections [31].

Comparisons with platelet-rich plasma (PRP) or extracorporeal shockwave therapy (ESWT) demonstrated similarly favorable short-term outcomes; however, BoNT showed significantly superior long-term results. It should be noted, however, that these findings are based on a limited number of studies, often involving only a single trial with a small patient population.

Researchers remain optimistic regarding the role of botulinum toxin (BoNT) in the management of chronic or refractory plantar fasciitis, with evidence demonstrating both statistically and clinically significant improvements in pain and function in the short term (within the first 6 months) and sustained benefits for up to 12 months [32].

The mechanisms underlying the clinical effects of BoNT in plantar fasciitis are only partially understood. BoNT inhibits the release of pain mediators—such as glutamate, substance P, and calcitonin gene-related peptide—at the presynaptic junction, through a mechanism analogous to its blockade of acetylcholine responsible for its paralytic effect. Although this analgesic mechanism would be expected to be transient, research has shown that its effects may persist for up to 12 months, far exceeding the duration predicted by the reversible neuromuscular blockade alone [33].

Moreover, BoNT appears to reduce peripheral sensitization, which subsequently mitigates central sensitization, thereby providing a plausible explanation for its prolonged analgesic effect. This pathophysiological rationale supports the use of intrafascial BoNT administration in the management of plantar fasciitis.

Injection of BoNT into the short plantar muscles induces temporary paralysis and reduces tissue tension, as these muscles insert onto the plantar fascia. The calcaneal (medial) and plantar (medial and lateral) branches of the posterior tibial nerve may become entrapped between tense anatomical structures; BoNT-induced muscle relaxation may alleviate this neurogenic pain.

BoNT injection into the gastrocnemius–soleus complex has also demonstrated efficacy, likely due to the anatomical and biomechanical continuum involving the gastrocnemius–soleus complex, calcaneus, and plantar fascia.

Variations in the methods used to target the affected tissue may account for differences observed between studies. Accurate localization of the anatomical structures ensures proper delivery of the substance and minimizes the risk of adverse effects. The plantar fascia can be localized through palpation or, more accurately, under ultrasound guidance. Similarly, muscle injections performed with ultrasound guidance or electrical stimulation allow for precise substance administration.

The analgesic effect of BoNT has been documented in several fascial pain syndromes, including lumbar, cervical, and shoulder fascial pain [33].

The safety of BoNT injections were documented through the mentioned studies. Researchers found no adverse effects, apart from pain at the injection site that disappeared in the day after therapy, one study reported mild numbness in the foot, a reaction common both for BoNT and corticosteroid groups [18]. There was no clinically significant reduction in muscle strength.

When compared with corticosteroid injections, BoNT provides similar short-term pain relief and functional improvement but yields significantly superior long-term outcomes. However, in isolated studies, platelet-rich plasma (PRP) demonstrated greater efficacy in one year, while extracorporeal shockwave therapy (ESWT) showed superior results at one month.

**Figure 3:**
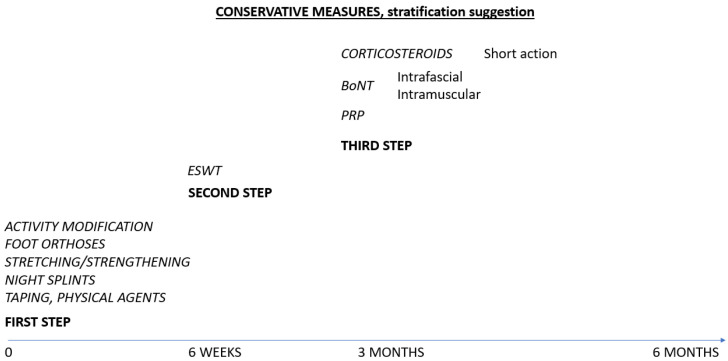
Suggested therapies for plantar fasciitis.

In the aim of integrating BoNT therapy in the management of chronic or relapsing plantar fasciitis, we designed the following scheme, as in Figure 3.

This study has some limitations. The number of RCT was small, the formulas of BoNT varied between studies, the route of administration was different, the target tissues were not always properly identified (as in palpatory guidance injection). BoNT in chronic plantar fasciitis remains an off-label therapy. There is also a small number of studies comparing different injectables therapies or injectables with ESWT. Stratification of therapies is still a challenge for actual guidelines.

An important issue is the target tissue for BoNT injection, as the mechanisms of action are different. Intrafascial injection is based on local analgesic effect, presumably by inhibiting substance P and other pain mediators from the presynaptic vesicles. Muscular injection implies a transient paralysis of muscles, either short plantar muscular to may compress local nerves or may tension the plantar fascia, or calf muscles based on the biomechanical synergy between triceps surae, Achilles tendon and plantar fascia. A clinical examination should orient the clinician to a contracture of specific muscles.

## 4. Conclusions

Botulinum toxin (BoNT) has emerged as an effective therapeutic option for chronic or refractory plantar fasciitis, resulting in both short- and long-term improvements in pain and function. Various administration techniques have been described, including intrafascial, intramuscular, and combined injections. In the short term, BoNT demonstrates comparable efficacy to corticosteroid injections, while in the long term it provides superior outcomes with a lower risk profile. Further studies are warranted to clarify its comparative effectiveness against platelet-rich plasma (PRP) and extracorporeal shockwave therapy (ESWT).

BoNT injection should be prescribed to selected patients with chronic or refractory forms of plantar fasciitis. A comprehensive clinical examination should reveal the tightening of surrounding structures—such as short plantar muscles, the Achilles tendon, and the gastrocnemius–soleus complex—in order to associate local administration for analgesic purposes with muscle infiltration for relaxing effects.

The administration of BoNT should be reserved for chronic cases with the failure of conservative measures, including patient education, footwear alteration, physical therapy, and oral agents. Patients should follow physical exercises, especially stretching, and adapt their life-style conditions to minimize stress of the plantar fascia.

## Figures and Tables

**Table 1:** Studies selected for the research.

Study, Year of Publication	Type of Study, No of Patients Duration of Symptoms	Study Design	Outcomes	Moments of Study	Results	Observation
Babcock, 2005 [11]	Prospective, randomized, double-blind, placebo-controlled study, 43 feet, 6 months	Group A: 70 UI ona-BTX-A, (40 UI intrafascial, 30 UI intramuscular) Group B: placebo Palpatory guidance	Pain (VAS) and pain relief (VAS) Pressure algometry Function (Maryland Foot Score)	Baseline, 3 weeks, 8 weeks	Group A: 3 and 8 weeks: significant pain and functional improvement	Treatment target: plantar fascia and the underlying muscles (adductor hallucis, flexor digitorum brevis) No adverse effects
Peterlein, 2012 [12]	Prospective, placebo-controlled, double-blind, phase II study of the efficacy and safety of a single injection of BoNT-A, 33 pts, 4 months	AbobotulinumtoxinA, 200 UI, versus saline, palpatory guidance, into the origin of the plantar fascia	Pain (VAS) Pressure algometry	2, 6, 10, 14, 18 weeks	Improvement in pain and function for the BoNT group but not in the significant range	No adverse effects
Huang, 2010 [13]	Prospective, randomized, placebo controlled, 50 pts, 3 months	50 UI onaBoNT-A versus saline, Ultrasound guided injection into the plantar fascia	Pain (VAS) Fascia thickness and fat and thickness (ultrasound) COP * velocity Foot pressure	Baseline, 3 weeks, 3 months	VAS and plantar fascia thickness significantly reduced at 3 weeks and 3 months COP velocity significantly increased at both moments Fat pad thickness unchanged	No adverse reaction
Ahmad, 2017 [14]	Prospective, randomized, controlled, 50 pts, 18.8 weeks (6–40 weeks)	Incobotulinum 100 UI into FDB * (EMG guidance) versus saline (placebo)	Pain (VAS) Function (FAAM)	6 months, 12 months	At 6 and 12 months, BoNT provided significant improvement in pain and function comparative with saline	No adverse reaction
Abbasian, 2019 [15]	Randomized, placebo controlled, 32 pts, 10 weeks	BoNT (70 IU) versus placebo (saline) into the medial head of gastrocnemius, ultrasound guided	Pain (VAS) Function (AOFAS, ability to fully bear weight, gastrocnemius strength, time to return to work, and the ability to wear normal shoes) ROM dorsiflexion Satisfaction (Likert scale)	Baseline, 1, 3, 6, and 12 months	Pain and function improved significantly in both groups at 1 year, with better results in BoNT group.	No effects on muscle strength
Diaz-Llopis, 2011 [16]	Prospective, randomized, single blind trial, 56 pts, 6 months	OnaBoNT-A 70 UI (Babcock method) versus corticosteroid (CS) + local anesthetic Palpatory guidance	Foot Health Status Questionnaire	1 and 6 months	1 month: BoNT and CS equally efficient 6 months: significant better result for BoNT	no adverse reaction
Samant, 2018 [17]	Prospective, randomized, controlled, 50 pts, 3 months	BoNT (100 UI) Corticosteroids US-guidance into the fascia	Pain (VAS) Fascia thickness (US)	Baseline, 1 and 3 weeks, 3, 6 and 12 months	Intra-group: significant improvement in pain. BoNT better results at 12 months on pain and fascia thickness	No adverse reaction
Geeta, 2022 [18]	Prospective, randomized, 100 pts (3 months)	BoNT (50 UI BoNT-A) CS Palpatory guidance in the most painful point on the medial aspect of the heel	Pain (VAS) Function (FAAM)	Baseline, 1, 2, 4 and 6 months	Pain and function improved in both groups, CS up to 4 months and declined, BoNT persisting up to 6 months	Mild numbness in foot in both groups in a small number of patients
Ruiz-Hernández, 2024 [19]	Randomized, 59 pts (3 months)	BoNT PRP	Pain (VAS), function (AOFAS, FAAM) Fascia thickness (US)	Baseline, 1, 2 and 12 months	1 months: BoNT significantly better results on pain 12 months: PRP significant better results	No adverse reaction
Elizondo-Rodríguez, 2020 [20]	Randomized, controlled, double-blind, 71 pts (2 months)	Group 1: local anesthetic Group 2: CS Group 3: 200 UI aboBoNT-A Ultrasound guided	Pain (VAS) Function (Maryland Foot Score) Fascia thickness Ankle ROM	2 weeks, 1, 3 and 6 months	All parameters improved in all groups at 6 months, no significant difference between groups	No side effects
Ahadi, 2022 [21]	Prospective, randomized, single blind, 35 pts (3 months)	Group A: CS into the fascia Group B: BoNT in flexor digitorum brevis and quadratus plantae US-guidance	Pain (VAS) Function (Foot and Ankle Ability Measures) Fascia thickness (US)	Baseline, 3 and 12 weeks, 6 months	Intragroup: all patients improved significantly at 3 weeks Group A: the results vanished over time Group B: sustained results up to 12 weeks and maintained at 6 months	No adverse reaction
Elizondo-Rodriguez, 2013 [22]	Prospective, randomized, double blind, controlled, 36 pts, 14 months	Group A: BoNT (250 UI triceps surae) Group B: CS (into the plantar fascia, palpatory guidance) All groups: stretching exercise	Pain (VAS), Function (Maryland Foot and Ankle scale, AOFAS, FADI)	Baseline, 15 days, 1, 2, 4 and 6 months	15 days: both groups improved From 1 month, BoNT group has significant better improvement in pain and function	No adverse reaction
Roca, 2016 [23]	open label, prospective, randomized study, 72 pts, 6 months	Focused ESWT, one session BoNT-A (onaBoNT) Babcock technique	Pain (VAS, Roles, and Maudsley scale) Quality of life (EQ-5D) Function (Foot Health Status Questionnaire) Plantar fascia thickness (ultrasound)	Baseline, 1, 2 months	Intragroup: significant improvement in pain and function Intergroup: significant better results for ESWT group	no adverse reaction
Placzek, 2005 [24]	Prospective, open, uncontrolled, case series, 9 pts, 4 months	BoNT (aboBoNT-A) 200 UI subfascially in four different directions through one injection Palpatory guidance	Greatest pain (last 48 h, VAS) Pain at rest (last 48 h, VAS) Muscle force Pain progression	Baseline, weeks 2, 6, 10, 14, 26, 39, and 52	Pain reduction at 2 weeks, persisting up to 14 weeks and visible at 52 weeks	No muscle weakness
Radovic, 2020 [25]	Longitudinal, prospective, pilot study on 4 patients with Plantar Heel Pain Syndrome (3 months–3 years)	AboBoNT in abductor hallucis (50 UI) and quadratus plantae muscle (50 UI) under electrical stimulation	Pain (VAS) Function (FAAM) Disability (PFPS)	Baseline, 1 week, 3 weeks, 6 weeks, 12 weeks, and 26 weeks	significant improvement at 6 weeks and 24 weeks	No muscle atrophy or loss of foot structure
Chou, 2011 [26]	Case report, 8 months	BoNT, 70 UI (40 units in the tender region of the heel medial to the base of the plantar fascia insertion, and 30 units in the most tender point of the foot arch (between a point about 1 in anterior to the heel and another point at the middle of the foot)	Pain (VAS) Pressure algometry Ultrasound exam	Weeks 1, 3, 5, 7	Pain and PPT decreased from 1 to 3 and 5 weeks and increased slightly at 7 weeks (still lower than the baseline) Fascia thickness decreased	
Diaz-Llopis, 2013 [27]	Observational, 24 pts	Same study group as Diaz-Llopis, 2011 [16]		12 months	Continued improvement, not significant	

* FDB, flexor digitorum brevis; FAAM, Foot and Ankle Ability Measures; VAS, visual analog scale; CS, corticosteroids; AOFAS, American Orthopaedic Foot and Ankle Society; FADI, Foot and Ankle Disability Index; ROM, range of motion; EQ-5D, European Quality of Life scale; COP, centre of pressure; PFPS, Plantar Fasciitis Pain/Disability Scale; US, ultrasound.

**Table 2:** Doses and routes of administration of BoNT formulas.

Target Structure	Onabotulinum Toxin A	Abobotulinum ToxinA	Inobotulinum Toxin A
Fascia, at the origin	40–50 UI	100–200 UI	100 UI
Short plantar muscles (quadratus plantae, abductor hallucis, flexor digitorum brevis, flexor hallucis brevis)	30 UI	50 UI	NA
Gastrocnemius—soleus complex	70 UI medial gastrocnemius	100 UI medial gastrocnemius 100 UI lateral gastrocnemius 50 UI soleus	NA

## Data Availability

No new data was created.
